# Melatonin as an anti-stress signal: effects on an acute stress model and direct actions on interrenal tissue in goldfish

**DOI:** 10.3389/fendo.2023.1291153

**Published:** 2024-01-08

**Authors:** Clara Azpeleta, Mª Jesús Delgado, Juriaan R. Metz, Gert Flik, Nuria de Pedro

**Affiliations:** ^1^ Departamento de Genética, Fisiología y Microbiología, Unidad Docente de Fisiología Animal, Facultad de Ciencias Biológicas, Universidad Complutense de Madrid, Madrid, Spain; ^2^ Departamento de Medicina, Facultad de Ciencias Biomédicas y de la Salud, Universidad Europea de Madrid, Madrid, Spain; ^3^ Department of Animal Ecology and Physiology, Radboud Institute for Biological and Environmental Sciences (RIBES), Radboud University, Nijmegen, Netherlands

**Keywords:** food intake, locomotor activity, cortisol, HPI axis, ACTH, CRH, stress, melatonin

## Abstract

**Background:**

Melatonin is a key hormone in regulation of circadian rhythms, and involved in many rhythmic functions, such as feeding and locomotor activity. Melatonin reportedly counteracts stress responses in many vertebrates, including fish. However, targets for this action of melatonin and underlying mechanisms remain unknown.

**Results:**

This study reports potential anti-stress properties of melatonin in goldfish *(Carassius auratus)*, with a focus on its effect on plasma cortisol, food intake, and locomotor activity, all of them involved in the responses to stress exposure. Indeed, acute injection of melatonin counteracted stress-induced hypercortisolinemia and reduced food intake. The reduced locomotor activity following melatonin treatment suggests a possible sedative role in fish. To assess whether this anti-stress effects of melatonin involve direct actions on interrenal tissue, *in vitro* cultures of head kidney (containing the interrenal cortisol-producing tissue) were carried out in presence of ACTH, melatonin, and luzindole, an antagonist of melatonin receptors. Melatonin *in vitro* reduced ACTH-stimulated cortisol release, an effect attenuated by luzindole; this suggests the presence of specific melatonin receptors in interrenal tissue.

**Conclusions:**

Our data support a role for melatonin as an anti-stress signal in goldfish, and suggest that the interrenal tissue of teleosts may be a plausible target for melatonin action decreasing cortisol production.

## 1 Introduction

The regulation of food intake in fish is a complex process involving an interplay of several central and peripheral signals, mainly of metabolic and neuroendocrine character (1–3). One of the main brain centers involved in such regulation is the hypothalamus, which integrates all these signals, and responds with the release of neuropeptides that participate in feedback loops responsible for appetite and energy balance regulation. The hypothalamus also integrates the stress response in the generation of adaptive changes in food intake and energy expenditure according to the exposure to stressful conditions (1, 3). Under stressful conditions, the regulatory mechanisms governing food intake in fish become disrupted, affecting the expression of appetite-related neuropeptides. This, in turn, alters the hypothalamic integration of orexigenic and anorexigenic signals, leading to changes in food intake (4, 5).

Stress has been shown to have a negative impact on food intake in fish (4, 6–9). The activation of hypothalamus-pituitary-interrenal (HPI) axis implies the recognition of a stress stimulus and the consequent activation of hypothalamus, which releases corticotropin releasing hormone (CRH) that, in turn, stimulates the synthesis and release of adrenocorticotropic hormone (ACTH) from the anterior pituitary gland. The ACTH binds to the melanocortin-2 receptor in the steroidogenic interrenal cells in the head kidney (containing the fish analogue of the mammalian adrenal gland) and stimulates the signaling cascade of synthesis and release of cortisol (10, 11). Since the HPI axis is activated in response to stress in fish, components of this axis (CRH, ACTH and cortisol) may be involved in the food intake reduction induced by stress exposure in teleosts (4, 12). Locomotor activity is also one of the behavioural parameters that show changes after stress exposure (13). However, it is difficult to set a general conclusion of this effect, as published results in fish show a variety of responses depending on the type of stressor, the time of the day fish are exposed to the stressor or the behavioural characteristics of the fish (13–15).

Melatonin (MEL) is a neurohormone mainly synthetized in pineal gland and involved in several physiological activities in vertebrates including fish, playing a role in the adaptive behavior of the animal to the environment (16–19). As a key signal in the regulation of circadian rhythms, MEL is related to many functions that have in common a rhythmic expression, such as feeding and locomotor activity (16, 19). Regarding feeding regulation, MEL is known to act as an anorexigenic signal, since this hormone generally reduces food intake in teleosts after both acute (20–22) and chronic treatments (20, 23–25). This anorectic effect can be mediated by other feeding regulators, since MEL stimulates anorexigenic and inhibits orexigenic signals (23, 26). Although MEL administration reduces locomotor activity in fish (20, 27, 28) and promotes a sleep-like state in zebrafish (*Danio rerio*) (29, 30) and the threespot wrasse (*Halichoeres trimaculatus*) (31), it has been suggested that the anorectic effect of MEL goes beyond the sedative action of this hormone on locomotor activity (16). On the other hand, a feeding reduction similar to that produced by exogenous MEL (21) did not modify locomotor activity in this species (27), suggesting an independence of feeding and locomotor activity responses to MEL administration.

An interesting interplay between MEL and stress has been suggested in fish (32), since this hormone can attenuate some stress responses, such as the increase in plasma cortisol levels and the food intake inhibition (28, 33–36). Previous reports on mammals and birds have also shown that MEL modulates the activity of the hypothalamus-pituitary-adrenal (HPA) axis, counteracting the glucocorticoid elevations induced by stress (37–41). Evidence in mammals suggests that MEL can affect the HPA axis at both the central and peripheral level (42): MEL exerts central actions at hypothalamic level (43) and direct actions on the adrenal gland inhibiting ACTH-stimulated glucocorticoid production (44–46). Nonetheless, the precise mechanisms by which MEL affects the synthesis and release of cortisol in fish under stressful conditions have not been entirely elucidated.

This study addresses the potential anti-stress properties of MEL in goldfish, a commonly used model in comparative research (47). To achieve this, we employed a 1 min air exposure protocol, widely used as a model of acute stress in fish (13), for examining its effects on circulating cortisol levels, food intake, and locomotor activity, which are some of the most typical responses to stress. Additionally, we examined potential targets in the HPI axis where MEL can exert its anti-stress effects. We tested the possible effects of MEL on circulating cortisol *in vivo* after pharmacological stimulation of the HPI axis at different levels, *viz*. the hypothalamus, pituitary gland, and interrenal tissue. To further explore the possible direct effects of MEL on interrenal tissue, we conducted *in vitro* cultures of the head kidney (including interrenal tissue) in presence of ACTH, MEL, and luzindole, the general antagonist of MEL receptors.

## 2 Materials and methods

### 2.1 Animals and maintenance

Goldfish were obtained from a commercial supplier in Madrid (ICA, Spain). Fish were maintained in 60-l aquaria, with constant flow of filtered tapwater, and fed daily at 10:00 h with floating pellets (1% body weight, bw, Sera Biogram). Photoperiod was 12L:12D (lights on at 08:00 h) and water temperature was 21 ± 1°C. Fish were kept under these conditions for at least 15 days before the experiments; fish showed normal feeding and activity patterns during this period. All procedures complied with the Guidelines of the European Union Council (UE63/2010) and the Spanish Government (RD53/2013) for the use of animals in scientific research and were approved by the Animal Experimentation Committee of Complutense University and the Community of Madrid (PROEX 107/20).

### 2.2 Hormone administration *in vivo*

ACTH (1-39, CAS n° 9061-27-2) and CRH (1-41, CAS n° 86784-80-7) were dissolved in teleost saline (20 mg Na_2_CO_3_ per 100 ml of 0.6% NaCl (21)). MEL (CAS n° 86784-80-7) was previously dissolved in ethanol (5% v/v). All hormones were purchased to Sigma-Aldrich (Spain). Fish were anesthetized in water containing tricaine methanesulphonate (MS-222, 0.14 g/l; Sigma-Aldrich, Spain) and injected at 10:00 h. Intracerebroventricular (ICV) injections were carried out using a 0.3 mm Microlance needle connected to a 5 μl Hamilton microsyringe with an 18 Venocath cannula. The ICV injections were performed through the central junction between the parietal and frontal bones. Intraperitoneal (IP) injections were carried out with a 1 ml syringe and 0.3 mm Microlance needle, close to the ventral midline posterior to the pelvic fins. Hormone doses and injection volumes (10 µl/g bw for IP and 1 µl/fish for ICV administration) were previously established: MEL (21), CRH (48, 49). ACTH dose was estimated by performing a prior dose-response experiment (50–200 ng/g; unpublished data), choosing the minimum dose that significantly increased cortisol.

### 2.3 Experimental designs

#### 2.3.1 Effect of IP administration of MEL on stress response

Goldfish (31.9 ± 1.1 g) were divided into the following four groups (n=8/group):

- Control + saline: fish were immersed into an anesthetic dose of MS-222 (0.14 g/l), and IP injected with teleost saline.
- Stress + saline: fish were exposed to acute stress (1 min of air exposure), and during that minute, they were IP injected with teleost saline.
- Stress + MEL (2 µg/g): fish were exposed to acute stress, and IP injected with MEL (2 µg/g bw).
- Stress + MEL (20 µg/g): fish were exposed to acute stress, and IP injected with MEL (20 µg/g bw).

Blood samples from the caudal vessels were collected from anaesthetized fish (MS-222, 0.14 g/l) at 30- and 120- min post-injections using a 1 ml sterile plastic heparinized syringe and a 0.5 mm Microlance needle. Plasma obtained by centrifugation was stored frozen at -80°C until cortisol assay. Food intake was quantified at 2 h post-injection (see 2.5 below). Locomotor activity was recorded (see 2.6 below) for 48 h (24 h before and 24 h after treatments).

#### 2.3.2 Effect of IP administration of MEL on CRH- and ACTH-stimulated cortisol

In the first experiment, goldfish (10.1 ± 0.5 g) were divided into four groups (n=8/group) and received two injections separated 30 min, as follows:

- Control: IP teleost saline followed by ICV teleost saline.
- CRH: IP teleost saline followed by ICV CRH (0.1 µg/g bw).
- MEL: IP MEL (20 µg/g bw) followed by ICV teleost saline.
- MEL + CRH: IP MEL (20 µg/g bw) followed by ICV CRH (0.1 µg/g bw).

In the second experiment, goldfish (30.35 ± 0.97 g) were divided into four groups (n=8/group) and received two IP consecutive injections, as follows:

- Control: two injections with teleost saline.
- ACTH: first injection with teleost saline and the second with ACTH (50 ng/g bw).
- MEL: first injection with MEL (20 µg/g bw) and the second one with teleost saline.
- MEL + ACTH: first injection with MEL (20 µg/g bw) and the second one with ACTH (50 ng/g bw).

In both experiments, blood samples were collected twice, immediately before the injections (t = 0) and 120 min afterwards, and were managed and stored as stated in 2.3.1.

#### 2.3.3 Effect of MEL on ACTH-stimulated cortisol release in cultured head kidneys

Goldfish (35.8 ± 1.7 g, n=6/group) were sacrificed by overdose MS-222 (0.28 g/l), and each head kidney was carefully dissected, rinsed in saline medium and placed into culture chambers. Medium (NaCl 128 mM, KCl 2mM, CaCl_2_.2H_2_O 2 mM, glucose 0.25%, BSA 0.03%, ascorbic acid 0.1 mM, HEPES 0.015 M, pH 7.4) was saturated with carbogen (5% CO_2_:95% O_2_) and kept at 22°C. A superfusion culture system (flow rate 30 µl/min) was used as described by Metz and coworkers (50), and the outline of the protocol is shown as **Supplementary Figure 1**. After 150 min of stabilization, tissues were incubated in the presence of different MEL doses (1, 10 and 100 nM) during 75 min (interval 150-225 min). ACTH (50 nM) was added to the medium during 15 min (180-195 min interval). Eluted fractions were collected in 5- or 15- min intervals and stored at -80°C until cortisol analysis.

To determine the specificity of the MEL effect, we used luzindole (LUZ, 1 µM; Sigma Chemical, Spain), a general competitive antagonist of membrane-bound MEL receptors. Head kidneys were removed from goldfish (67.5 ± 2.3 g) and cultured under similar conditions to those above mentioned. With the information obtained in the superfusion culture, a static culture system was used (51) and outlined as in **Supplementary Figure 2**. After 120 min of stabilization period, four experimental groups (n=10/group) were established:

- Control: head kidneys were incubated in culture medium alone for 120 min.
- ACTH: head kidneys were incubated for 60 min in culture medium alone plus 60 min stimulated with ACTH (50 nM)
- ACTH+MEL: head kidneys were incubated for 30 min in culture medium, 30 min with MEL (10 nM) and 60 min with ACTH (50 nM) and MEL (10 nM)
- ACTH +MEL+LUZ: head kidneys incubated for 30 min with LUZ (1 µM), 30 min with LUZ (1 µM) + MEL (10 nM) and the last 60 min with ACTH (50 nM) + LUZ (1 µM) + MEL (10 nM).

Supernatants were collected at different time points and stored at -80°C until cortisol analysis.

### 2.4 Cortisol analysis

Plasma cortisol levels were determined by ELISA (Demeditec, Germany), previously validated for goldfish plasma (27, 52).

### 2.5 Food intake quantification

Food in excess (4% bw) was delivered immediately after the treatments, and food intake (FI) was individually measured at 2 h post-injection as reported (48). FI = Wi – (Wf x F), where Wi and Wf are the dry weight of initial and final food, respectively, and F (correction factor) = 1.16 represents the reduction in food weight by dissolution in water, calculated as previously described (48).

### 2.6 Locomotor activity determination

Locomotor activity was individually recorded in 5-l aquarium (19 cm length, 19 cm width, 14 cm height) from 24 hours before until 24 hours post-injection (48 hours in total). Fish were transferred to each individual tank 24 hours before any experimental procedure. Locomotor activity was registered following a protocol previously described (53). Briefly, one infrared photocell (Omron Corporation, E3S-AD12, Japan) was fixed onto the aquaria wall. Photocells constantly emitted an infrared light beam. Each time fish swam in that zone, the infrared light was interrupted, generating an output signal. The number of light beam interruptions was automatically counted and stored every 10 min with an actimeter and data-acquiring software Adq16 (Micronec, Spain). To avoid visual interferences during the experiment, aquaria walls were covered with opaque paper.

### 2.7 Statistical analysis

Data are represented as mean + standard error of the mean (SEM). Normality and homoscedasticity of data was confirmed by the Shapiro-Wilk and Levene tests, and data were adjusted to a logarithmic or square root scale if necessary. Statistical differences among groups were determined by one-way (sections 2.3.3, superfusion culture) or two-way ANOVA (sections 2.3.1, 2.3.2 and 2.3.3, static culture) followed by multiple comparison test Student-Newman-Keuls (SNK), using the Statgraphics software. Differences were considered significant when p<0.05.

## 3 Results

### 3.1 Effect of IP administration of MEL on stress response

**Figure 1:**
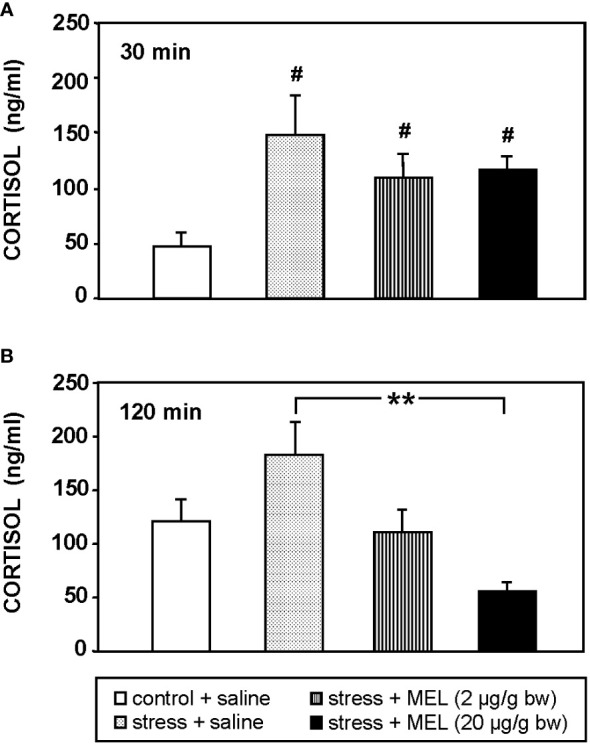
Cortisol plasma levels in stressed and IP MEL-injected goldfish (2 and 20 µg/g bw) at **(A)** 30, and **(B)** 120 min post-injection. Data are expressed as mean + SEM (n=8/group). #: p<0.05, respect to control group; **: p<0.01.

The air exposure for 1 min is a valid model of acute stress in fish that increased plasma cortisol levels (p<0.05) 3-fold compared to unstressed fish, at 30 min after stress exposure (**Figure 1A**). The increase in plasma cortisol lasted in stressed fish for at least 120 min post-stress, although no statistically significant differences were found compared to control group at this time (**Figure 1B**). The two doses of MEL tested (2 and 20 µg/g bw) counteracted the increase in plasma cortisol levels in stressed fish (**Figure 1B**), reducing circulating cortisol four-fold by the administration of 20 µg/g (p<0.01). This effect was observed at 120 min (**Figure 1B**).

**Figure 2:**
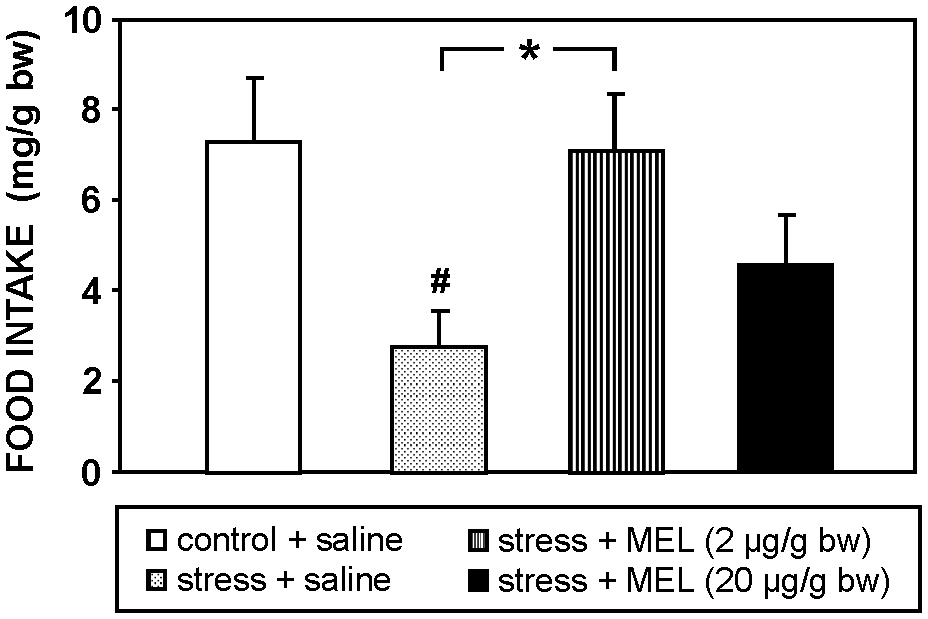
Effect of MEL administration (IP, 2 and 20 µg/g bw) on food intake at 2 h post-injection in goldfish exposed to an acute stress. Data are expressed as mean + SEM (n=8/group). #: p<0.05, respect to control group; *: p<0.05.

This acute stress diminished significantly (p<0.05) food intake compared to unstressed fish (**Figure 2**), and MEL injection (2 µg/g bw) totally counteracted this anorectic effect induced by stress. This reversal was only partial when MEL was injected at the higher dose (20 µg/g bw).

**Figure 3:**
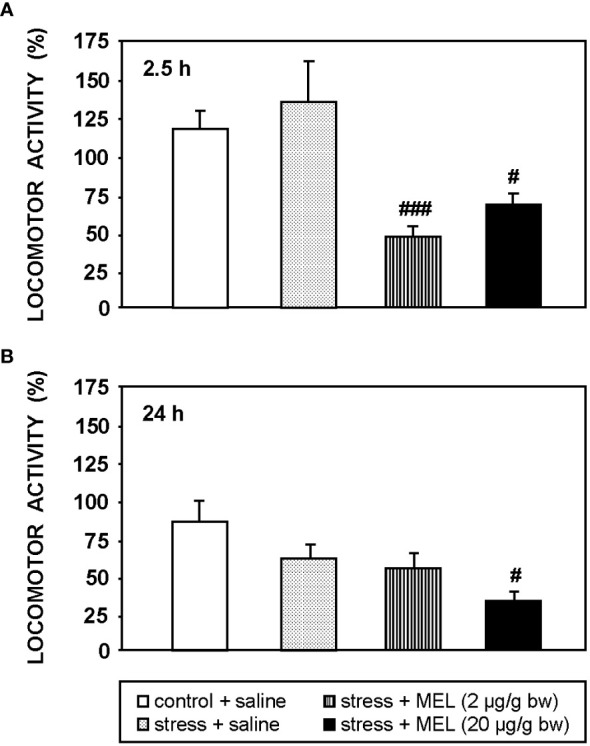
Locomotor activity at 2.5 h **(A)** and 24 h **(B)** post-injection in goldfish exposed to an acute stress and IP administration of MEL (2 and 20 µg/g bw). Data (mean + SEM, n=8/group) are expressed as a percentage respect to the activity registered during the same time interval before the treatments. #: p<0.05, ###: p<0.001, respect to control group.

The **Figure 3** shows the effect of an acute stress and MEL injection on locomotor activity at 2.5 and 24 h post-treatments. The acute stress produced by the exposure to air for 1 min did not modify significantly locomotor activity of goldfish at any of the two times studied, i.e. 2.5 and 24 h. The two doses of MEL reduced locomotor activity compared to both, control and stressed fish (p<0.001, 2 µg/g; p<0.05, 20 µg/g bw) at 2.5 h post-injection (**Figure 3A**). This effect of MEL on activity persists for at least 24 h at the highest dose (p<0.05; **Figure 3B**).

### 3.2 Effect of IP administration of MEL on CRH- and ACTH-stimulated cortisol

**Figure 4:**
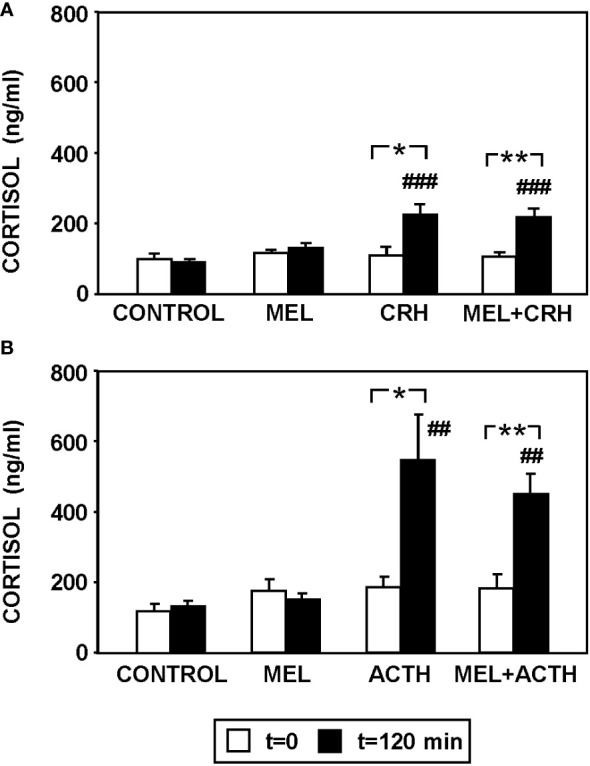
Effect of MEL administration (IP, 20 µg/g bw) on plasma cortisol levels in goldfish injected with **(A)** CRH (ICV, 0.1 µg/g bw) and **(B)** ACTH (IP, 50 ng/g bw) at 120 min post-injections. Data are expressed as mean + SEM (n=8/group). ##: p<0.01, ###: p<0.001, respect to control group; *: p<0.05, **: p<0.01.

**Figure 4** summarizes plasma cortisol levels in fish injected with MEL alone or combined with CRH (**Figure 4A**) or ACTH (**Figure 4B**). Circulating cortisol was similar in all fish at the beginning of the study (t=0). CRH injection significantly increased (p<0.001) circulating cortisol at 120 min post-injection, which was not modified by the pre-treatment with MEL (**Figure 4A**). Similarly, MEL administration did not counteract the increase in plasma cortisol levels induced by ACTH injection (**Figure 4B**).

### 3.3 Effect of MEL on ACTH-stimulated cortisol release in cultured head kidneys

**Figure 5:**
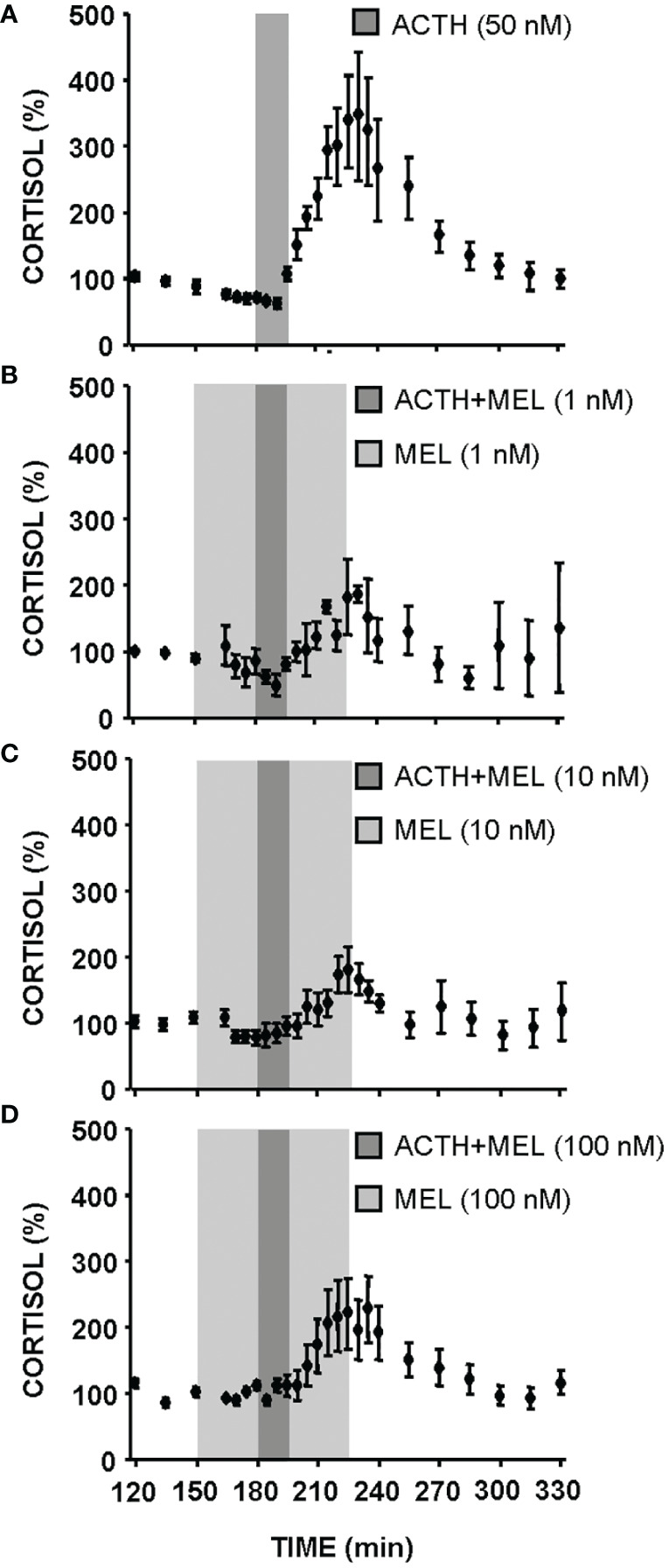
Cortisol release from goldfish head kidneys superfused with ACTH (50 nM) alone **(A)** or combined with MEL: 1 nM **(B)**, 10 nM **(C)**, and 100 nM **(D)**. Data (mean ± SEM, n=6/group) represent the percentage of cortisol release at each supernatant collection interval (5 or 15 min intervals) with respect to basal values (120-150 min time period). Dark grey bars represent incubation time in the presence of ACTH; light grey zone indicates incubation time in the presence of different concentrations of MEL (1, 10 and 100 nM).

**Figure 6:**
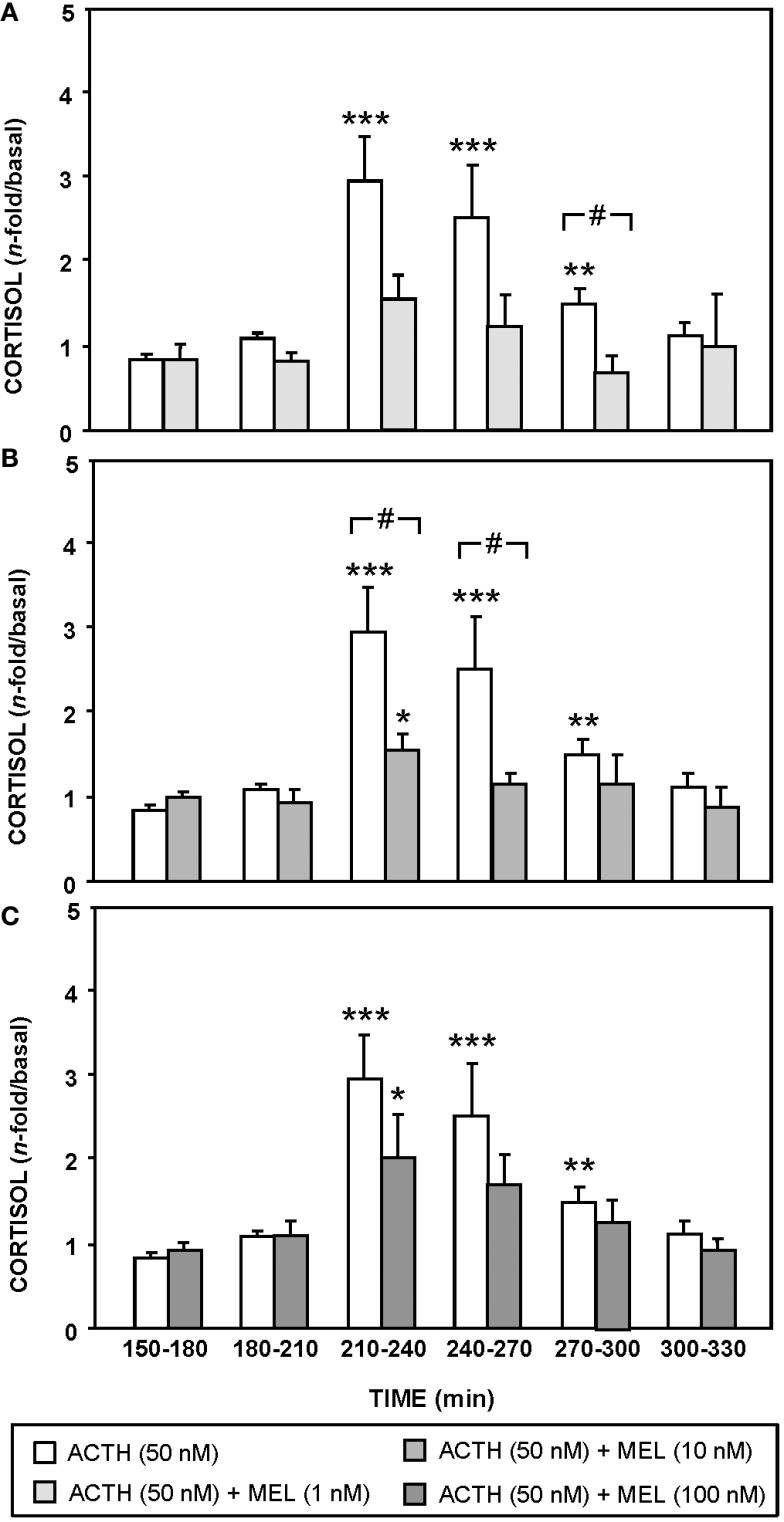
Effect of MEL on cortisol release from goldfish head kidneys stimulated with ACTH (50 nM, control group). This group is compared with MEL: 1 nM **(A)**, 10 nM **(B)**, and 100 nM **(C)** Data (mean + SEM, n=6/group) represent the increase of cortisol release respect to basal levels (120-150 min time period) in intervals of 30 min. *: p<0.05, **: p<0.01, ***: p<0.001, respect to basal levels within the same experimental group; #: p<0.05, among groups within the same time interval.

The effect of ACTH (50 nM) and MEL (1, 10 and 100 nM) addition on cortisol release by cultured goldfish head kidney is shown in **Figures 5**, **6**. The presence of ACTH in the culture medium highly increased cortisol release by the tissue (~3-fold respect to basal levels), with the highest stimulation in the interval of 210-270 min (p<0.001). This stimulation by ACTH was decreasing, being only 1.5 fold in the following interval (270-300 min), and basal cortisol levels were recovered at 300 min (**Figures 5A**, **6**). The pre-incubation with MEL (1 and 10 nM) attenuated the ACTH stimulation, by reducing the maximum release of cortisol to almost half of stimulation observed with ACTH alone during the 210-270 min interval (**Figures 5B, C**, **6A, B**). This counteracting effect on the ACTH-stimulated cortisol was not statistically significant in the experiment with the highest dose (100 nM) of MEL (**Figures 5D**, **6C**). Recovery of basal cortisol levels after ACTH stimulation is achieved earlier in MEL-treated head kidneys, at 240 min with the 1 nM dose, and at 255 min with the 10 nM dose.

**Figure 7:**
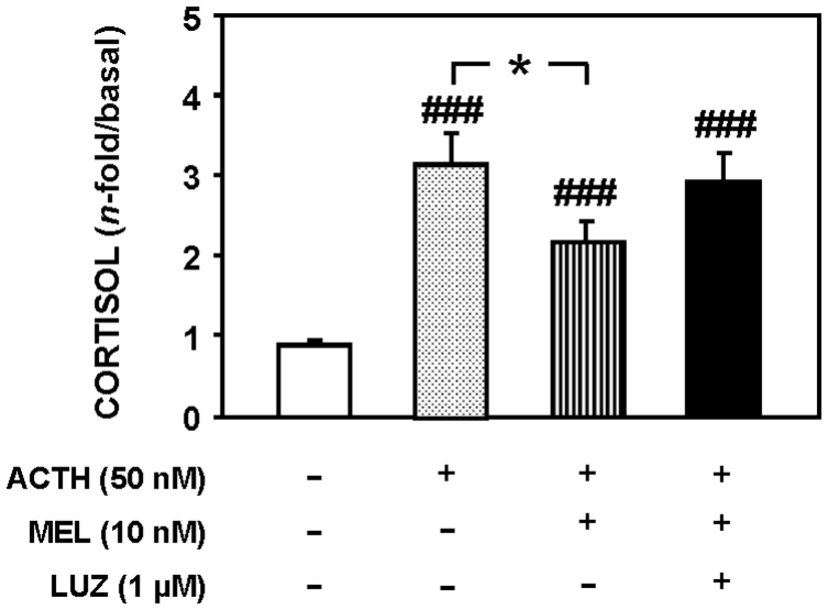
Cortisol released from goldfish head kidneys cultured in the presence of ACTH (50 nM), MEL (10 nM) and LUZ (1 µM). Data represent the increase of cortisol release (180-240 min) respect to basal levels (60-120 min). Data are expressed as mean + SEM (n=10/group). ###: p<0.001, respect to control group; *: p<0.05.

**Figure 7** shows cortisol release in a static culture of goldfish head kidneys incubated with ACTH (50 nM), MEL (10 µM) and the MEL antagonist, LUZ (1 µM). The presence of LUZ in the culture medium blocked the inhibitory effect of MEL (p<0.05) on cortisol release induced by ACTH (p<0.001).

## 4 Discussion

This study shows that MEL effectively counteracts hypercortisolinemia and anorexia following acute stress in goldfish and support an anti-stress role for this hormone in this teleost. Moreover, our findings imply that the interrenal tissue of teleosts is target for MEL action to decrease cortisol production.

The anorectic effect induced by acute stress here observed in goldfish has been previously reported in other fish species (7, 9, 54, 55). The variation in the physiological response to stress appears to be dependent on the type of stressor and its intensity (5) and also depends on the species (11, 56). Even so, several reports point to CRH and cortisol as the main neuroendocrine signals of the HPI axis involved in stress-induced anorexia. An anorectic effect after CRH administration has been described in a variety of fish species (12, 48, 57, 58). This anorectic action of CRH can be mediated by an interaction with the hypothalamic catecholaminergic systems (4, 49, 59, 60). Moreover, it has been suggested that melanocortin system is also involved, since stress increases MSH levels, which inhibits food intake in fish (4, 59). Several studies have also reported a reduction in food intake after cortisol treatment in fish (61–65), probably by modulation of some feeding regulator peptides, such as leptin and ghrelin (66, 67), suggesting that cortisol can participate in the regulation of food intake under stressful conditions. However, we cannot rule out that activation of the HPI axis modifies other neuropeptides involved in the feeding regulation (4).

One of the most relevant findings of this study is the reversion of stressful responses (food intake reduction and cortisol increase) by MEL injection in goldfish, supporting the role as anti-stress hormone also described in mammals (42, 68, 69). Our results agree with previous studies in other teleost species that reported a calming effect of this neurohormone under essentially stress-free and stressful conditions. Thus, MEL treatment reduced plasma cortisol levels in unstressed fish: goldfish (27); sea bass, *Dicentrarchus labrax* (70); Atlantic salmon, *Salmo salar*, and Coho salmon, *Oncorhynchus kisutch* (71). Besides, MEL attenuated cortisol increased by acute or chronic stress exposure in Senegalese sole (*Solea senegalensis*) (33, 35), rainbow trout (*Oncorhynchus mykiis*) (34), and zebrafish (28). The reversal of stress-induced anorectic effect by MEL in goldfish is also supported by previous results in rainbow trout (34) and brown trout (*Salmo trutta*) (72) treated with either MEL or its precursor tryptophan. Nevertheless, different feeding responses to MEL may also be attributed to the dose used. On one hand, the lower dose of MEL (2 µg/g bw) totally reversed the stress-induced decrease in food intake in goldfish, in agreement with the absence of anorectic effect at this dose in the same species (21). On the other hand, an anorectic effect was observed with higher doses (20 and 200 µg/g bw) of MEL (21), which could explain why the 20 µg/g dose of MEL used in the present study only partially restored food intake in stressed goldfish. Consequently, food intake in stressed goldfish administered with the highest dose of MEL might be influenced by a disturbed equilibrium between the stress-alleviating properties and the appetite-suppressing function of this hormone. Moreover, it has been suggested that the role of this hormone in the feeding regulation may vary depending on whether the animals are under basal or stressed conditions and their feeding status. In fact, MEL reduced food intake and increased hypothalamic serotonin and dopamine levels in non-stressed rainbow trout, while it reversed the feeding inhibition and the increase in serotonergic and dopaminergic activity induced by stress in this species (34). These differential effects of MEL on monoaminergic system in basal or stress conditions were also described in Senegalese sole (35). These data of MEL also support the idea that appetite signals under stress conditions do not function in the same way as they do under control conditions (4).

The relaxant effect of MEL reducing motor activity has been found in stressed (28) and present results; and unstressed fish (20, 27, 31). It is broadly accepted that MEL promotes sleep in vertebrates, and particularly in fish. Zhdanova and co-workers (29, 30) suggested that MEL promotes a sleep-like state in zebrafish. In support of MEL effects on stress alleviation, it has been also reported that this hormone mitigates other stress responses in fish, including those related to digestion, skin health, immune system function, and osmotic balance (18, 36, 73, 74). All these data support the hypothesis of a role of melatonin as a recovery or alleviating hormone in fish under environmental stressors (74). In this sense, a recent study has also revealed that MEL ameliorates silver nanoparticles-induced toxicity effects in Nile tilapia, *Orechromis niloticus* (75). It has even been described that MEL is able to improve the disorders induced by caffeine in the gut microbiota and brain neurotransmitter secretion of zebrafish (76).

Our results suggest that anti-stress effect of MEL can be mediated at different levels of the HPI axis. Bearing in mind the ability of MEL to cross the blood brain barrier (17), it is expected that this hormone reach neural sites of the HPI axis. Injections of CRH or ACTH increased cortisol in goldfish, which was not reversed by MEL pre-treatment. However, melatonin inhibited CRH-stimulated cortisol in birds (41). One possible explanation is that HPI axis pharmacological stimulation with these neuropeptides produced much greater activation than the physiological stimulation induced in the stress model by air exposure, masking the anti-stress effect of MEL. Another hypothesis is that MEL could be exerting its anti-stress effect at the hypothalamic level, inhibiting CRH production, thus rendering it ineffective when administered to fish with a HPI axis previously stimulated by exogenous administration of CRH or ACTH. Indeed, the stress-induced increase in hypothalamic CRH expression was attenuated by MEL injection in rainbow trout (35). This effect may be mediated by the serotonergic system, as in fish is known that serotonin agonists increase CRH expression in the hypothalamus (77), and MEL reverses the stress-induced hypothalamic increase in serotonergic activity (35). Additionally, we cannot exclude the involvement of other components of the CRH system, such as CRH receptors or CRH-binding protein (CRH-BP), also involved in stress regulation (78).

Our results in goldfish show that MEL directly regulates cortisol production in ACTH-stimulated head kidney, in agreement with previous studies in adrenal gland of mammals (44–46). The MEL action appears to be mediated through interaction with the G-protein coupled membrane bound MEL receptors, since the luzindole (a general antagonist of MEL receptors) counteracted, at least in part, the inhibitory effect of MEL on ACTH-stimulated cortisol production. This result is supported by MEL binding sites and mRNA expression of MEL receptors described in the head kidney of different teleosts (79–81). The mechanisms underlying the direct action of MEL on biosynthesis and/or release of glucocorticoids remain unknown, but some evidences in fish suggest the involvement of steroidogenic acute regulatory protein (STAR), as MEL also attenuated the stress-induced increase of expression of STAR mRNA in the head kidney in Senegalese sole (35) and goldfish (82). Nevertheless, it should also be noted that cortisol increase in fish is not always associated with an increase in STAR (35, 83), suggesting that alternative mechanisms might be targeted by MEL.

The physiological relationship between MEL and cortisol can also be discussed in the context of the functioning of the circadian system. Daily profiles of both hormones are considered robust outputs of the circadian system in fish (84). Daily profiles of MEL have been described in fish, with higher levels at night than during the light period (19). However, these daily profiles are not so clear for cortisol, and depend on the species, photoperiod, season and feeding and activity patterns (84). Additionally, the goldfish head kidney is considered a functional circadian clock (52, 85) and the effect of MEL could be related with its synchronizing role, although more information is still necessary to determine whether MEL is in fact an input that synchronises the interrenal clock and cortisol rhythms in fish.

The mechanism of MEL secretion during stress is an interesting and little studied topic. Pineal gland expresses glucocorticoid receptors in fish, and cortisol inhibits MEL synthesis by activating these receptors (86, 87). It is reported that cortisol directly activates glucocorticoid-responsive elements in the promoter of AANAT (arylalkylamine N-acetyltransferase, rate-limiting enzyme of MEL synthesis) (86). This result agrees with the reduction in MEL levels and AANAT activity in both, stressed and cortisol-treated fish (88, 89). Altogether these results are consistent with the anti-stress role described for this hormone. However, some works described increases in MEL levels induced by stressful conditions in fish (90) and mammals (91), which can be linked to different factors such as species, daytime of MEL measurement, duration of stress or type of stressor.

More experiments are still necessary to dilucidate aspects such as the molecular mechanisms by which MEL is exerting these effects, or its dependence on light/dark cycle. But altogether, our findings indicate that MEL can mitigate negative effects of stress in goldfish, and this suggests a high degree of conservation of this role as anti-stress hormone throughout phylogeny. In addition, we propose that the head kidney of goldfish could be one of the potential targets of MEL, where this hormone can act on (one or more of its) specific receptors to effectively reduce plasma cortisol levels. Hence, this possible role of melatonin as an anti-stress signal, together with his widely known role as a synchronizer of biological rhythms, results important for animal welfare, and particularly valuable in the field of aquaculture.

## Data availability statatement

The raw data supporting the conclusions of this article will be made available by the authors, without undue reservation.

## Ethics statement

The animal study was approved by Animal Experimentation Committee of the Complutense University of Madrid (PROEX 107/20). The study was conducted in accordance with the local legislation and institutional requirements.

## Author contributions

CA: Formal analysis, Investigation, Methodology, Writing – original draft, Writing – review & editing. MD: Conceptualization, Funding acquisition, Project administration, Supervision, Writing – original draft, Writing – review & editing. JM: Investigation, Methodology, Supervision, Writing – review & editing. GF: Investigation, Methodology, Supervision, Writing – review & editing. NP: Conceptualization, Funding acquisition, Project administration, Supervision, Writing – original draft, Writing – review & editing.
