# Supplementary material for: Melatonin as an anti-stress signal: effects on an acute stress model and direct actions on interrenal tissue in goldfish
Source: Front Endocrinol (Lausanne). 2024 Jan 8;14:1291153. doi: 10.3389/fendo.2023.1291153 (PMC10800973; doi:10.3389/fendo.2023.1291153)
Supplement: Supplementary file 1 [file DataSheet_1.pdf]

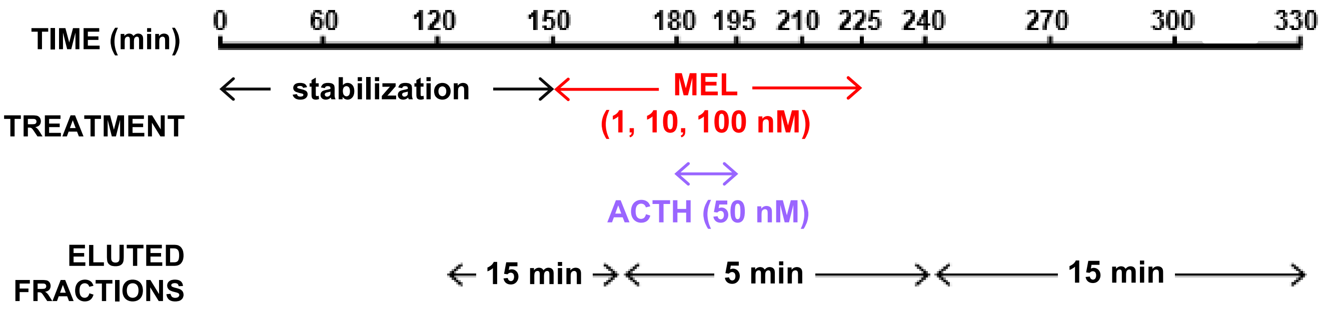

Supplementary Figure 1. Treatments and temporal sequence of flow through culture system in goldfish head kidney.

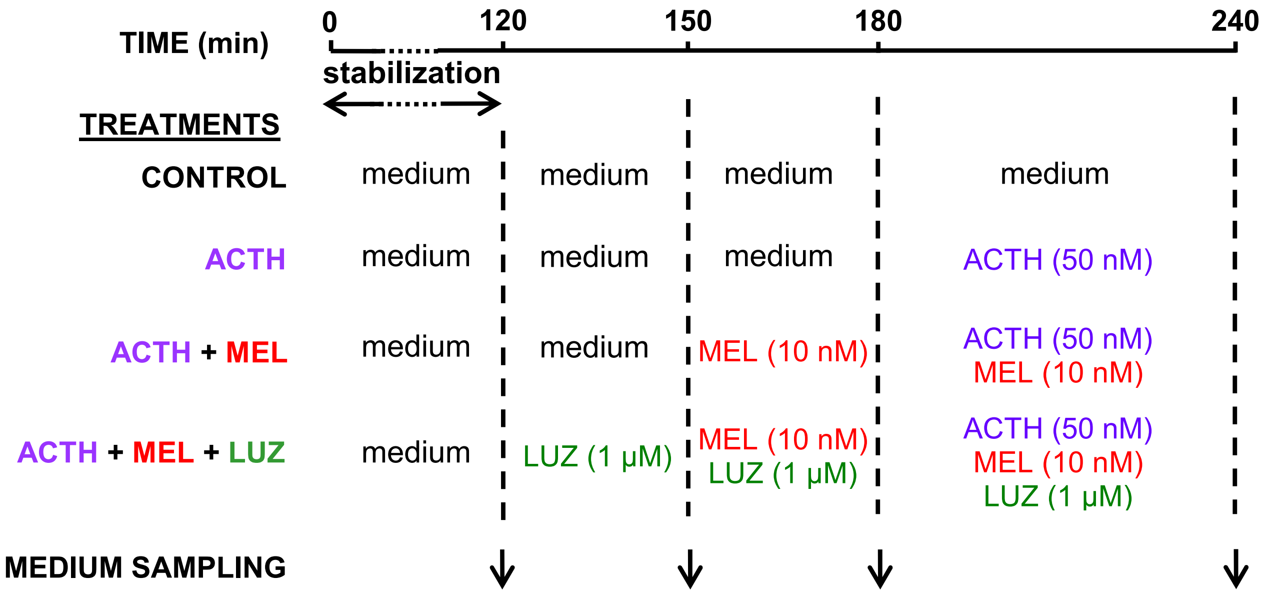

Supplementary Figure 2. Treatments and temporal sequence of static culture system in goldfish head kidney.
